# A Novel Position Estimation Method Based on Displacement Correction in AIS

**DOI:** 10.3390/s140917376

**Published:** 2014-09-17

**Authors:** Yi Jiang, Shufang Zhang, Dongkai Yang

**Affiliations:** 1 Information Science and Technology College, Dalian Maritime University, Dalian 116026, China; E-Mail: sfzhang@dlmu.edu.cn; 2 School of Electronic and Information Engineering, Beihang University, Beijing 100083, China; E-Mail: edkyang@buaa.edu.cn

**Keywords:** positioning method, displacement correction, time of arrival, automatic identification system (AIS)

## Abstract

A new position estimation method by using the signals from two automatic identification system (AIS) stations is proposed in this paper. The time of arrival (TOA) method is enhanced with the displacement correction, so that the vessel's position can be determined even for the situation where it can receive the signals from only two AIS base stations. Its implementation scheme based on the mathematical model is presented. Furthermore, performance analysis is carried out to illustrate the relation between the positioning errors and the displacement vector provided by auxiliary sensors. Finally, the positioning method is verified and its performance is evaluated by simulation. The results show that the positioning accuracy is acceptable.

## 1. Introduction

Robust position, navigation and timing (PNT) information is an essential foundation of e-navigation, developed by the International Maritime Organization (IMO), and intended to enhance marine navigation. Though the global navigation satellite system (GNSS) is the primary navigation system in maritime applications, an alternative position system to complement the existing GNSS may improve the robustness of the whole positioning and navigation process [1]. In fact, various position estimation methods and their applications in marine navigation have been widely investigated in the literature, such as e-Loran systems [2–5], inertial navigation systems [6,7], terrain referenced navigation systems [8], vessel traffic service and coastal surveillance systems [9,10], *etc*. But the automatic identification system (AIS), which is essentially a communication system [11], is seldom used for robust PNT. It provides position information obtained from GNSS. Obviously, it could promote a robust PNT for marine navigation if AIS could provide PNT information by itself, since vessels are mandated to carry AIS equipment according to the IMO requirements for its member countries [12]. To develop positioning service for AIS with widespread distribution is helpful to make use of the existing resources for enhancing robustness in marine navigation [13].

The main difficulty in obtaining the position information from AIS itself is that the number of received signals from AIS base stations may be fewer than necessary to solve the vessel's position, since AIS is originally designed as a communication system, not a PNT system. In other words, the geometrical distribution of AIS base stations may be poor for positioning. Actually, the placement of base stations provides sufficient signal coverage overlap for ensuring AIS reliability and stability. This implies that the vessel can generally receive signals from two base stations. In these cases, the traditional time of arrival (TOA) method is hard to use.

This paper presents a method based on displacement vector correction to estimate a vessel position by measuring signals from two AIS base stations. A vessel's position and its clock bias are estimated by the continuous range measurements in adjacent moments. The relationship of position information between the adjacent moments can be derived by the displacement vector. A displacement vector is calculated according to the heading and velocity provided by auxiliary sensors. Consequently, the position information is obtained by solving a system of four equations with three unknowns. Utilizing the existing devices already equipped in vessels as the auxiliary sensors, such as compasses, log indicators, will not increase the cost of the vessel navigation system. Thus the proposed method can maximize the reuse of resources to meeting IMO requirements for robust PNT information.

The rest of the paper is organized as follows: Section 2 discusses the TOA positioning method in AIS. The novel positioning method using displacement correction is presented in Section 3, including its mathematical model, implementation scheme and performance evaluation. In order to validate the proposed method, several simulations are provided in Section 4. Finally, some conclusions are put forth in Section 5.

## 2. TOA Positioning Method in AIS

**Figure 1.**
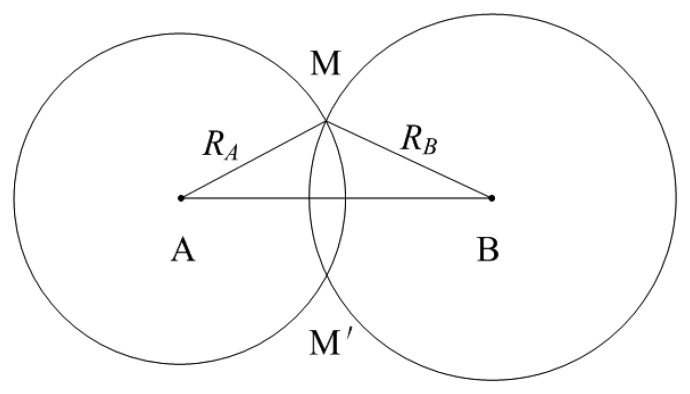
Geometrical principle of the TOA metohd.

Generally speaking, position estimation can be determined using the TOA technique [14,15]. Figure 1 shows the geometrical principle of the TOA method. The vessel lies on circles with radii R_1_ and R_2_ centered on the base stations (B_1_ and B_2_), whose position can be obtained precisely. By measuring the signals from at least two base stations, the position of the vessel can be determined by the intersection point of the circles. Though there exist two solutions M and M′, M′ can be easily excluded according to the approximate position of the vessel.

The latitude and longitude coordinates of the vessel are denoted by (*φ*,*λ*). For convenience, the changes in latitude and longitude are denoted by the vertical and horizontal increments (Δ*φ*,Δ*ω*). Then the updated latitude and longitude coordinates of the vessel (*φ*′,*λ*′) and (Δ*φ*,Δ*ω*) satisfy the following equations:

(1) $$\begin{array}{l} \varphi^{\prime }=\varphi +\Delta \varphi \\ \lambda^{\prime }=\lambda +\Delta \omega \text{sec}\varphi \end{array}\}$$

The position equation can be modeled as the function of the range *L* = *L*(*φ*,*ω*), that is generally nonlinear:

(2) $$L_{i}=\cos^{-1}\left(\sin\varphi \sin\varphi_{i}+\cos\varphi \cos\varphi_{i}\cos\left(\lambda -\lambda_{i}\right)\right)$$

where a subscript *i* denotes the *i*th AIS base station. The linear position equation using Taylor-series keeping only terms below second order is given by:

(3) $${\bar{L}}_{i}={\hat{L}}_{i}+\frac{\partial {\hat{L}}_{i}}{\partial \varphi }\delta \varphi +\frac{\partial {\hat{L}}_{i}}{\partial \omega }\delta \omega$$

where *L̄_I_* and *L̂_I_* are the measured and estimated ranges from the *i*th base station to the vessel, respectively. (δ*φ*,δ*ω*) are the corrections to the estimated position vector in the vertical and horizontal directions. The terms of partial derivative in Equation (3) can be calculated by the following formulas:

(4) $$\begin{array}{l} \frac{\partial {\hat{L}}_{i}}{\partial \varphi }=-\cos{\hat{\gamma }}_{i}\\ \frac{\partial {\hat{L}}_{i}}{\partial \omega }=-\sin{\hat{\gamma }}_{i} \end{array}\}$$

where *γ_i_* is the estimated azimuth angle directed from the estimation of the vessel's position to the *i*th base station. *γ*_i_ can be calculated as follows:

(5) $${\hat{\gamma }}_{i}=\tan^{-1}\left(\frac{\cos\varphi_{2}\sin\left(\lambda -\lambda_{i}\right)}{\cos\varphi \sin\varphi_{i}-\sin\varphi \cos\varphi_{i}\cos\left(\lambda -\lambda_{i}\right)}\right)$$

The above conclusions can be obtained in the situation that time can be synchronized between the vessel and AIS base stations. However this is a difficult task to achieve in reality. For AIS, Equation (3) can be rewritten as:

(6) $${\bar{L}}_{i}={\hat{L}}_{i}+\frac{\partial {\hat{L}}_{i}}{\partial \varphi }\delta \varphi +\frac{\partial {\hat{L}}_{i}}{\partial \omega }\delta \omega +c\delta t$$

where *δt* is the clock bias between the vessel and AIS base stations and *c* is the velocity of light in the free space. Thus the clock bias augments the two-dimensional position vector forming a three-dimensional state vector. To estimate the vessel position, more than three independent TOA measurements from different AIS base stations are required at the same time. However, the vessel can only receive signals from two base stations under most conditions in the existing AIS setup. Therefore, the vessel position estimation cannot be obtained using the traditional TOA positioning method.

## 3. Position Estimation Based on Displacement Correction

### 3.1. A Mathematical Model

At time *k* and time *k* + 1, the vessel locates at (*φ^k^*, *ω^k^*) and (*φ^k^*^+1^, *ω^k^*^+1^), respectively, which are to be determined. According to Equation (6), the linearized position equation at time *k* is:

(7) $${\bar{L}}_{i}^{k}={\hat{L}}_{i}^{k}+\frac{\partial {\hat{L}}_{i}^{k}}{\partial \varphi^{k}}\delta \varphi^{k}+\frac{\partial {\hat{L}}_{i}^{k}}{\partial \omega^{k}}\delta \omega^{k}+c\delta t$$

Here a superscript *k* is used to denote time *k*. If measurements from two different AIS base stations are obtained, Equation (7) can be written in matrix form as:

(8) $$\left[\begin{array}{c} \delta L_{A}^{k}\\ \delta L_{B}^{k} \end{array}\right]=\left[\begin{array}{ccc} \frac{\partial {\hat{L}}_{A}^{k}}{\partial \varphi^{k}} & \frac{\partial {\hat{L}}_{A}^{k}}{\partial \omega^{k}} & c\\ \frac{\partial {\hat{L}}_{B}^{k}}{\partial \varphi^{k}} & \frac{\partial {\hat{L}}_{B}^{k}}{\partial \omega^{k}} & c \end{array}\right]\;\left[\begin{array}{c} \delta \varphi^{k}\\ \delta \omega^{k}\\ \delta t \end{array}\right]$$

where $\delta L_{i}^{k}={\bar{L}}_{i}^{k}-{\hat{L}}_{i}^{k}$. The subscript *A* and *B* correspond to the two AIS base station in Figure 1, respectively. Similarly, the position matrix at time *k* + 1 is given by:

(9) $$\left[\begin{array}{c} \delta L_{A}^{k+1}\\ \delta L_{B}^{k+1} \end{array}\right]=\left[\begin{array}{ccc} \frac{\partial {\hat{L}}_{A}^{k+1}}{\partial \varphi^{k+1}} & \frac{\partial {\hat{L}}_{A}^{k+1}}{\partial \omega^{k+1}} & c\\ \frac{\partial {\hat{L}}_{B}^{k+1}}{\partial \varphi^{k+1}} & \frac{\partial {\hat{L}}_{B}^{k+1}}{\partial \omega^{k+1}} & c \end{array}\right]\;\left[\begin{array}{c} \delta \varphi^{k+1}\\ \delta \omega^{k+1}\\ \delta t \end{array}\right]$$

where (δ*φ^k^*^+1^, δ*ω^k^*^+1^) refer to the correction values between the true and estimated position of the vessel at time *k* + 1. There are five unknowns in four equations, thus it is insufficient to solve the position.

The estimate of relative displacement vector Δ*X^k^* = (Δ*φ^k^*, Δ*ω^k^*) relative to the location at time *k* can be calculated as follows:

(10) $$\begin{array}{l} \Delta \varphi^{k}=v^{k}\Delta T\cos\alpha^{k}\\ \Delta \omega^{k}=v^{k}\Delta T\sin\alpha^{k} \end{array}\}$$

where *v* and *α* are vessel's true velocity and heading, respectively; Δ*T* is a time interval between the adjacent moments. The real-time vessel's velocity and heading information can be obtained by auxiliary sensors. Thus, the positioning relationship between time *k* and time *k* + 1 is established using the displacement vector according to Equation (11):

(11) $$\begin{array}{c} {\hat{\varphi }}^{k+1}={\hat{\varphi }}^{k}+\Delta \varphi^{k}\\ {\hat{\omega }}^{k+1}={\hat{\omega }}^{k}+\Delta \omega^{k} \end{array}\}$$

According to the vessel's displacement vector between time *k* and *k* + 1, calculated by the auxiliary information, the correction values in two adjacent moments are equal, that is:

(12) $$\begin{array}{c} \delta \varphi^{k+1}=\delta \varphi^{k}\\ \delta \omega^{k+1}=\delta \omega^{k} \end{array}\}$$

Then the position matrix at time *k* + 1 can be rewritten as:

(13) $$\left[\begin{array}{c} \delta L_{A}^{k+1}\\ \delta L_{B}^{k+1} \end{array}\right]=\left[\begin{array}{ccc} \frac{\partial {\hat{L}}_{A}^{k+1}}{\partial \varphi^{k+1}} & \frac{\partial {\hat{L}}_{A}^{k+1}}{\partial \omega^{k+1}} & c\\ \frac{\partial {\hat{L}}_{B}^{k+1}}{\partial \varphi^{k+1}} & \frac{\partial {\hat{L}}_{B}^{k+1}}{\partial \omega^{k+1}} & c \end{array}\right]\left[\begin{array}{c} \delta \varphi^{k}\\ \delta \omega^{k}\\ \delta t \end{array}\right]$$

Thus, the position at time *k* + 1 is the function of the position at time *k*, eliminating two unknown variables from five unknown variables. The positioning matrix is the combination of positioning matrices in the adjacent moments, that is:

(14) $$\left[\begin{array}{c} \delta L_{A}^{k}\\ \delta L_{B}^{k}\\ \delta L_{A}^{k+1}\\ \delta L_{B}^{k+1} \end{array}\right]=\left[\begin{array}{ccc} \frac{\partial {\hat{L}}_{A}^{k}}{\partial \varphi^{k}} & \frac{\partial {\hat{L}}_{A}^{k}}{\partial \omega^{k}} & c\\ \frac{\partial {\hat{L}}_{B}^{k}}{\partial \varphi^{k}} & \frac{\partial {\hat{L}}_{B}^{k}}{\partial \omega^{k}} & c\\ \frac{\partial {\hat{L}}_{A}^{k+1}}{\partial \varphi^{k+1}} & \frac{\partial {\hat{L}}_{A}^{k+1}}{\partial \omega^{k+1}} & c\\ \frac{\partial {\hat{L}}_{B}^{k+1}}{\partial \varphi^{k+1}} & \frac{\partial {\hat{L}}_{B}^{k+1}}{\partial \omega^{k+1}} & c \end{array}\right]\;\left[\begin{array}{c} \delta \varphi^{k}\\ \delta \omega^{k}\\ \delta t \end{array}\right]$$

Four position equations at two adjacent moments can be obtained according to measurements. Moreover, there are three unknown variables to solve four equations in Equation (14). The correction values of the vertical and horizontal components and the clock bias can be calculated according to the above position matrix by a least squares estimation approach. Then the vessel's latitude and longitude at time *k* can be obtained according to Equation (1).

*Remark 1*: The combination of dead reckoning and range measurements is an existing approach which has been used in many fields such as GPS-INS [16,17], robotics [18,19], vehicular *ad-hoc* networks [20–22] and pedestrian localization in indoor environments [23,24]. Dead reckoning determines a present position from a known past position. However pure dead reckoning methods are prone to accumulated errors over time. Positioning methods based on range measurement have better accuracy, but are sometimes hard to implement or use due to the time synchronization issue. Many algorithms are used to combine dead reckoning and range measurements to improve the accuracy, such as a Kalman filter, a particle filter or a Markov method [25,26]. It should be noted that both dead reckoning and range measurements can estimate the position by themselves, as shown in the previous work. The combination of two methods can improve the accuracy. However, position estimation is impossible when a vessel receives signals from only two AIS base stations. The proposed position estimation method is investigated for this situation.

### 3.2. Implementation Scheme

**Figure 2.**
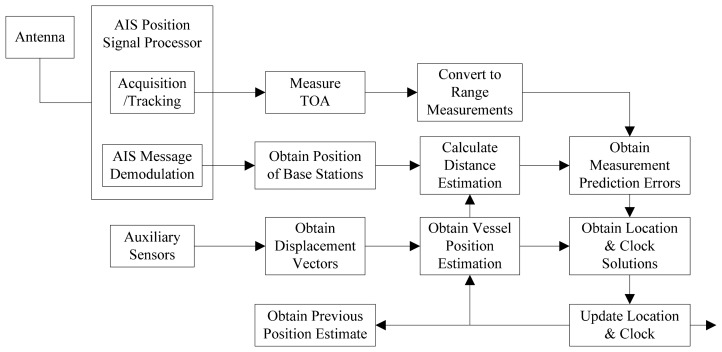
Implementation scheme of position estimation based on displacement correction.

The implementation scheme of the proposed position estimation method based on displacement correction is illustrated in Figure 2.

Firstly, signals from AIS base stations are received by the vessel's receiver via an antenna. Then a radio frequency (RF) front-end intercepts the incoming RF signal with 160 MHz and converts it to an appropriate intermediate frequency (IF) for digitization, e.g., 455 KHz. The digital IF signals are processed in the AIS position signal processor. It is organized into functionally identical channels, each dynamically assigned to a different AIS base station. The signals are acquired, tracked and messages are demodulated.

Secondly, according to the AIS signal tracking results, TOA measurements are produced to get the propagation time between the vessel and different AIS base stations. Then TOA measurement is used to calculate the range *L̅_i_*, which is called pseudorange, since bias exists in the vessel's clock. At the same time, the exact position information of each AIS base station is extracted from the demodulated messages.

Thirdly, the range ${\hat{L}}_{i}^{k}$ between the vessel and the different AIS base station at time *k* can be estimated, since the initial position estimation of the vessel and the exact coordinates of the AIS base stations are known. The initial position estimation of the vessel can use the vessel's position information at the last moment stored by the receiver or provided from external devices. Position information of AIS base stations can be obtained by the demodulated messages. The measured and estimated ranges are used to calculate the measurement prediction errors $\delta L_{i}^{k}$ at time *k* in the position matrix as Equation (8).

Then, the displacement vector (δ*φ^k^*^+1^, δ*ω^k^*^+1^) of the vessel is obtained from auxiliary sensors at time *k* + 1 according to Equation (10). The vessel position (*φ̂_k_*_+1_, *ω̂_k_*_+1_ at time *k* + 1 can be estimated according to Equation (11). Thus the ranges ${\hat{L}}_{i}^{k+1}$ between the vessel and the base stations at time *k* + 1 can be calculated. These estimated ranges and the TOA measurements at time *k* + 1 are used to construct the corresponding position equations as Equation (13). Finally, the vessel's position and clock bias can be solved by the combination of position equations at two adjacent moments according to Equation (14).

### 3.3. Performance Analysis

**Figure 3.**
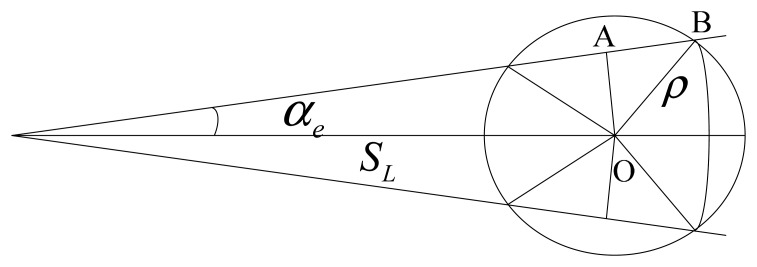
Positioning error influenced by heading and voyage error.

Positioning accuracy is one of the important technical parameters to evaluate position estimation method [27,28]. The positioning error is influenced by the errors of the displacement vector, including the heading error and the voyage error, as depicted in Figure 3.

As shown in Figure 3, *α_e_* is the heading error of the auxiliary sensor and *S_L_* is voyage determined by the vessel's velocity *v_K_* and the time interval Δ*T*. The positioning error caused by the heading error is given by:

(15) $$OA=\frac{\alpha_{e}S_{L}\pi }{180^{\circ }}=\frac{\alpha_{e}v_{k}\Delta T}{57.3^{\circ }}$$

The vessel voyage error is decided by the correction rate *v*_Δ*L*_ of the auxiliary sensor and *S_L_*. The positioning error caused by voyage error is:

(16) $$AB=v_{\Delta L}S_{L}=v_{\Delta L}v_{k}\Delta T$$

Thus the positioning error *ρ* caused by the heading error and voyage error can be calculated by Equation (17):

(17) $$\rho =\sqrt{OA^{2}+AB^{2}}=\sqrt{{\left(\frac{\alpha_{e}S_{L}}{57^{\circ }.3}\right)}^{2}+{\left(v_{\Delta L}S_{L}\right)}^{2}}$$

*Remark 2*: The vessel's position error calculated by Equation (17) is always larger than the actual error when the voyage is long, since the above conclusion is based on the condition that random errors are superimposed on each other, but in an actual situation, random errors may cancel each other out, so the actual positioning error may be less than the theoretically calculated error according to Equation (17). As the positioning error exists, the actual vessel position is in a circle with center at *O* and the radius of *ρ* shown in Figure 3. That is to say an error boundary of the vessel position is indicated by a 63.2%∼68.3% probability circle.

As already mentioned, *α* and *v* denote the vessel's true heading and velocity, respectively. The above analysis shows that the position errors are dependent on the measurement errors of the true heading and voyage. Considering the influence of wind and flow, errors exist in the heading and the velocity provided by the auxiliary sensors. Thus the error of vessel position caused by the wind and flow may come down to the heading error and the voyage error caused by auxiliary sensors. Our error analysis method is also applicable in this scenario.

## 4. Simulation Analysis

### 4.1. Signal Coverage Area of AIS Base Stations

**Figure 4.**
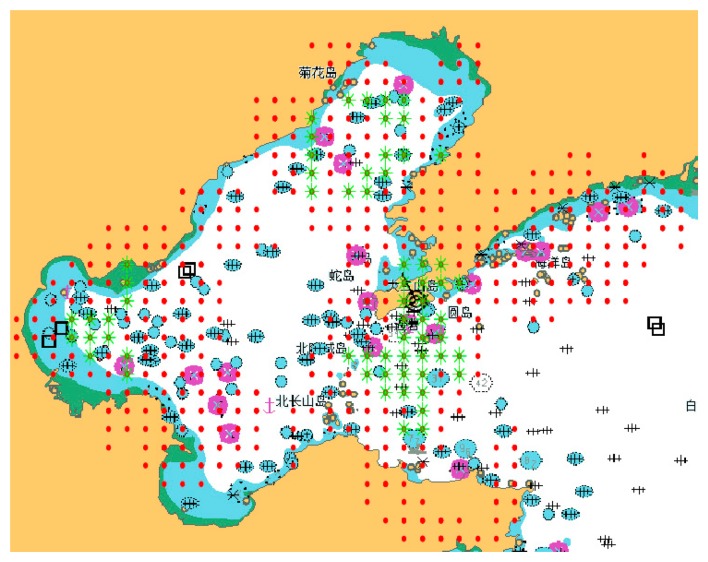
Signal coverage area of AIS base station in the North Sea region.

An AIS base station network has already been established by Chinese government, including a national AIS management center located in Tianjin, management centers for the three sea regions (North, South and East Sea), nineteen district management centers and nearly one hundred AIS base stations. The coastal areas in China are all within coverage areas. Let us take the North Sea region as an example. The positioning system using AIS is destined to be a regional radio navigation system limited to the very high frequency (VHF) range. Typical coverage is 25 nautical miles offshore. Figure 4 is the signal coverage area of the AIS base station in the North Sea region. The overlay signal coverage area of three AIS base stations is represented by the green “*”, while the overlay area of only two AIS base stations is represented by the red “.”. It can be seen that it is common for vessels to receive signals from two base stations in the actual AIS. Therefore, the position estimation proposed in the paper is very practical.

### 4.2. Performance Simulation

**Figure 5.**
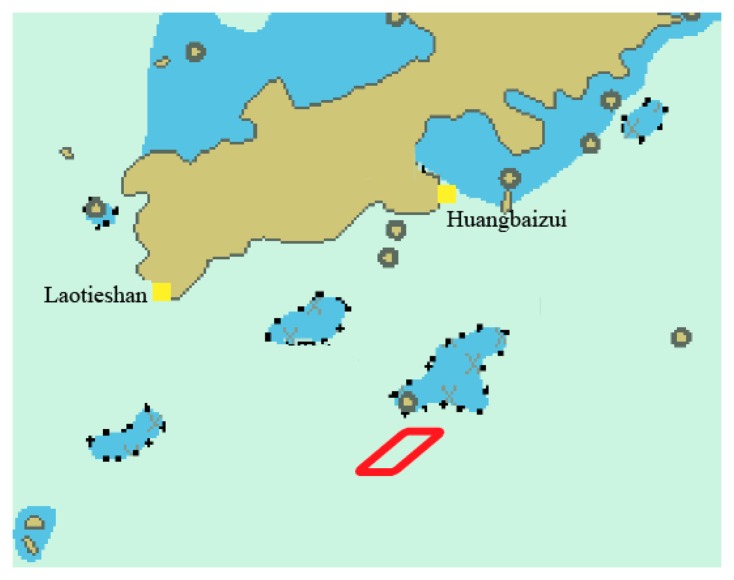
Distribution of AIS base stations and the vessel's trajectory.

This section presents simulations to demonstrate the proposed method and evaluate its performance. AIS base stations named Laotieshan and Huangbaizui are marked with yellow boxes in Figure 5. Table 1 gives information about these two AIS base stations, including the maritime mobile communications service identity (MMSI), the latitude and longitude coordinate. In the simulation scenario, the vessel's trajectory is a parallelogram racetrack. The initial position of the vessel is at (38°34.414′N, 121°38.187′E) with the velocity of 20 knots (10.2888 m/s) and heading of 90 degrees. After straight line motion for 500 s, the vessel will turn 45 degrees left with a centripetal acceleration of 0.2 m/s^2^. The vessel's trajectory is indicated by the red solid line in Figure 5.

**Figure 6.**
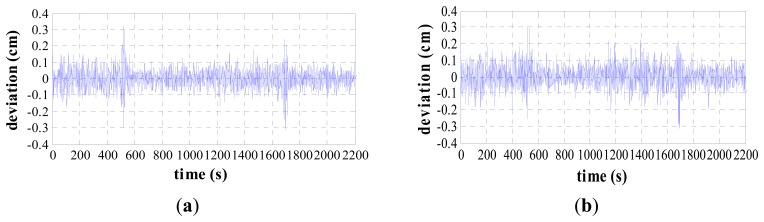

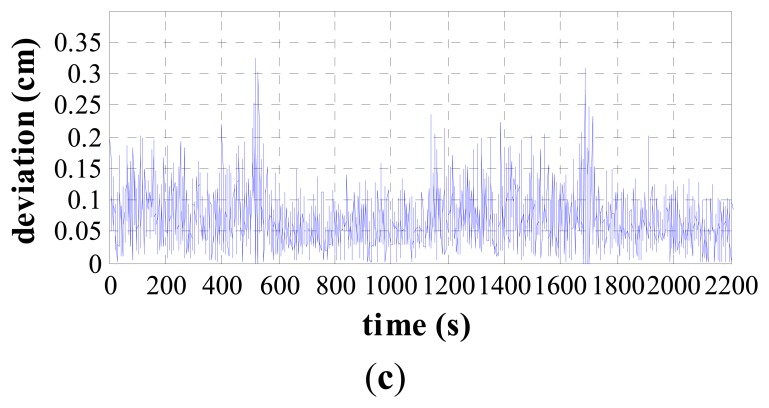
Error curves of vessel dynamic accuracy. (**a**) Latitude error. (**b**) Longitude error. (**c**) Positioning error.

Firstly, the proposed method is verified by assuming that the velocity and heading provided by auxiliary sensors are accurate. The impact of auxiliary sensor errors will be discussed later in Section 4.3. The deviations between the true and estimated vessel's position during the vessel's movement are compared in Figure 6, including the longitude, latitude and positioning error.

It can be seen from the simulation results that the standard deviation of the latitude error is 0.0541 cm, the standard deviation of the longitude error is 0.0574 cm and the standard deviation of the positioning error is 0.0698 cm. The estimated errors are all at the centimeter-level. The above simulation results confirm the validity and feasibility of the proposed method.

### 4.3. Auxiliary Sensor Error Simulation

Based on the above discussion, the proposed method provides centimeter-level positioning accuracy with the assumption that the velocity and heading are accurate. However, this assumption is hard to realize in engineering practice. The accurate displacement vector may not be obtained due to errors of the auxiliary sensor. The effects of the auxiliary sensor errors on the positioning accuracy are investigated in this section. The errors of auxiliary sensor including the heading and the voyage errors are discussed in relation to their impact on positioning accuracy.

The vessel's heading is provided by the compass. Here we use an Anschutz Gyro Compass Standard 22 in the simulation, whose static measuring error is within ±0.1° sec*φ* and dynamic measuring error is within ±0.4° sec*φ* [29]. Thus heading errors can be calculated when the vessels are on the equator, at latitude of 45° or at the poles by referring to Table 2.

**Figure 7.**
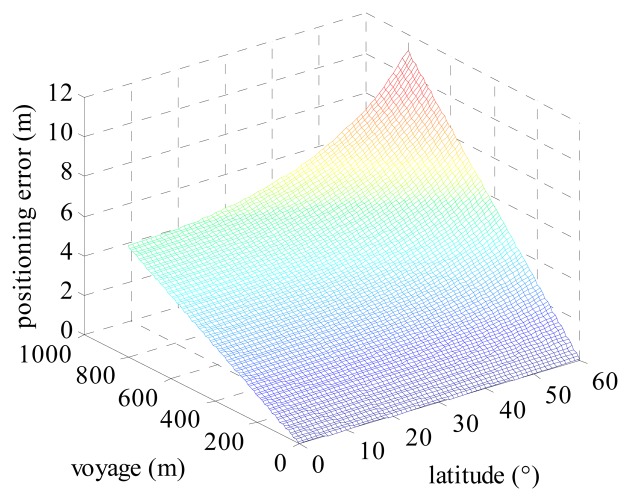
Positioning error influenced by heading error.

Positioning errors influenced by the heading error can be calculated by Equation (15). Positioning errors change with the latitude of the vessel and the voyage as depicted in Figure 7. It's shown that positioning errors increase with the increasing latitude and voyage. A vessel's voyage is determined by its velocity and duration of time. We take a FURUNO DS-80 as a log indicator in the simulation. The accuracy of the velocity is the greater one of 1.0 percent of the velocity and 0.1 knots [30]. In other words, when the vessel's velocity is greater than 10 knots, the accuracy of the log indicator is 1.0 percent of the velocity. Table 3 gives the velocity accuracy and error correction rate of the log indicator for a maximum velocity of 5, 10 and 26 knots, respectively. The error correction rate is equal to the accuracy divided by its corresponding velocity.

When the time interval is set at 1, 5, 10 and 60 s, the voyages are shown in Table 4.

Positioning errors *ρ* can be calculated by Equation (17), according to the heading error in Table 2, the log correction rate in Table 3 and the voyage in Table 4. The positioning error *ρ* varying with the velocity and time interval in the case of given heading error is shown below.

**Figure 8.**
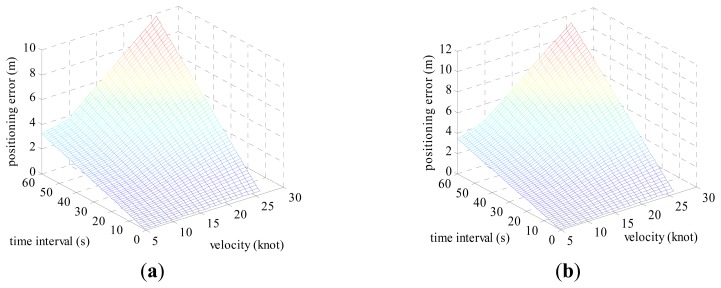
Positioning error influenced by displacement vector. (**a**) Heading error is 0.4°. (**b**) Heading error of 0.5659°.

The velocity varies from 5 to 26 knots and the time interval is set from 1 s to 60 s. The positioning error when the vessel is on the equator is shown in Figure 8a. Figure 8b shows the positioning error when the vessel locates at latitude 45°. It can be seen from the simulation results that the positioning error *ρ* is on the order of magnitude of 10 m using common vessel devices such as the Anschutz Gyro Compass Standard 22 and FURUNO DS-80 as auxiliary sensors.

## 5. Conclusions

As one of the widely used land-based communication systems, AIS can also provide range-mode positioning service to promote the robustness of the traditional navigation systems equipped on vessels. However, for the situations when the received signals using the usual TOA positioning method are not enough, the auxiliary sensors on the vessel provide additional motion information that can help to estimate the vessel's position. A new position estimation method based on displacement correction in AIS is proposed to determine the vessel's position when the signals can be received from only two AIS base stations. The mathematical analysis and simulation results show that the positioning accuracy of the new method is dependent on the accuracy of the sensors. Based on our implementation scheme, our future work will focus on the realization in a mobile AIS device. This will lay the foundation for the demonstration project using the new ranging-mode of AIS established in the coastal areas of China.

## Figures and Tables

**Table 1.** Information of the AIS base stations and mobile locations.

**Name**	**MMSI**	**Latitude**	**Longitude**
Laotieshan	4131101	38°43.6420′N	121°08.1330′E
Huangbaizui	4131104	38°54.2850′N	121°42.9500′E

**Table 2.** Heading accuracy.

**Position**	**Equator**	**45°**	**Pole**
Static error (°)	0.1000	0.1414	∞
Dynamic error (°)	0.4000	0.5659	∞

**Table 3.** Velocity accuracy and error correction rate.

**Velocity (knots)**	**5**	**10**	**26**
Accuracy (knots)	0.1	0.1	0.26
Error correction rate	2.0%	1.0%	1.0%

**Table 4.** Voyage with different time intervals.

**Time Intervals (s)**	**Velocity (knots)**		

	**5**	**10**	**26**
1	2.572222 m	5.144444 m	13.3755544 m
5	12.86111 m	25.72222 m	66.877772 m
10	25.72222 m	51.44444 m	133.755544 m
30	77.16666 m	154.33332 m	401.266632 m
60	154.33332 m	308.66664 m	802.533264 m
